# Early diagnosis and critical management of wound botulism in the emergency department: a single center experience and literature review

**DOI:** 10.1186/s12245-021-00375-4

**Published:** 2021-09-22

**Authors:** Michael M. Neeki, Fanlong Dong, Chuck Emond, Carol Lee, Arianna S. Neeki, Keeyon Hajjafar, Megan Messinger, Caitlyn O. Anderson, Reza Hajjafar, Rodney Borger

**Affiliations:** 1 Department of Emergency Medicine, Arrowhead Regional Medical Center, 400 N. Pepper Ave, Suite # 107, Colton, CA 92324 USA; 2 California University of Science and Medicine, San Bernardino, CA 92324 USA

**Keywords:** Wound botulism, Intravenous drug use, Intramuscular drug use, Skin popping

## Abstract

**Background:**

Clostridium botulinum remains a major threat to a select population of subcutaneous and intramuscular drug users. We conducted a retrospective study of patients who were diagnosed with wound botulism and their clinical presentations to the Emergency Department (ED).

**Results:**

A total of 21 patients met the inclusion criteria and all had a confirmed history of heroin use disorder. Initial presentation to the ED included generalized weakness (*n* = 20, 95%), difficulty swallowing (*n* = 15, 71%), and speech/voice problems (*n* = 14, 79%). Sixteen patients (76%) also presented with visible skin wounds and fifteen (71%) required mechanical ventilation (MV). Patients who presented with dysphagia as well as dysarthria and/or dysphonia were more likely to require a percutaneous endoscopic gastrostomy (PEG) tube. Patients who required MV and PEG tubes were noted to have a longer hospital length of stay (LOS) due to the severity of the disease progression.

**Conclusions:**

Emergency physicians should remain vigilant about early recognition of wound botulism, especially in patients who inject drugs.

## Introduction

Wound botulism is a life-threatening disease with rapidly worsening clinical course. In the USA, wound botulism is associated with subcutaneous injection of black tar heroin and has a mortality of 13.2% [1, 2]. The introduction of black tar heroin (BTH) in the 1990s led to an increase in non-intravenous injection, most often into the subcutaneous tissue (“skin popping”) and sometimes into muscle. Both methods require less accuracy than IV injection, remain viable after superficial venous access is exhausted, and result in slower absorption of the drug [1, 3]. BTH is still the predominant form of heroin in the USA, and California alone accounts for three-quarters of all wound botulism cases in the country [2]. This method of BTH administration and the associated likelihood of infection by *Clostridium botulinum* (*C. botulinum*) has increased morbidity and complications in individuals [1].

There are many opportunities to become contaminated with *C. botulinum* in the production and distribution process for BTH, and the spores will survive substances and temperatures that kill other bacteria. The drug is frequently cut with anything of a similar dark brown color, including dirt and wood pulp, which may introduce *C. botulinum* spores. Spores have also been found on drug paraphernalia [1]. Subcutaneous injection then creates an ideal anaerobic breeding ground for *C. botulinum* and the formation of botulinum neurotoxin [1, 2]. The sharing of contaminated needles is also common among a selected demographic of drug users and can contribute to wound botulism [4]. If an abscess develops after injection of BTH, the anaerobic environment allows spores to germinate and the organism to thrive [5]. *C. botulinum* neurotoxin quickly spreads systemically via the lymphatic and vascular systems [4]. Botulism progresses rapidly, and patients may present with bulbar and other nonspecific symptoms such as weakness, dysphagia, gait disturbance, and dyspnea [4].

Early diagnosis requires high clinical suspicion and can be confirmed by electrophysiological and laboratory studies. However, the average delay between blood sample acquisition and results of the toxin assay can be up to a few days [6, 7]. As a result, empiric antitoxin acquisition from the Centers for Disease Control and Prevention (CDC) and early administration is recommended as soon as the diagnosis is suspected clinically [7]. Since the antitoxin is only available from the CDC, physicians should anticipate a delay between their request and the arrival of antitoxin at the requesting facility [8]. As a result, acquiring the antitoxin from the CDC is the rate-limiting step to initiating adequate therapy [8]. Prolonged delay of appropriate and necessary treatment can result in rapid clinical deterioration of the patient and even death [9].

Due to the rarity of botulism and clinicians’ unfamiliarity with its clinical presentation, inclusion of the possibility of botulism in the differential diagnosis and subsequent treatment is often absent or delayed. Successful treatment of wound botulism is well known to be time dependent, and a delay in treatment may create a survival risk for the patient. This study aims to evaluate the most prevalent signs and symptoms of patients who presented to the emergency department (ED) with wound botulism and their course of treatment.

## Methods

This study was approved by the Institutional Review Board at Arrowhead Regional Medical Center (ARMC). ARMC is a 456-bed acute-care teaching facility located in Colton, California. ARMC is an American College of Surgeons verified Level II trauma center and one of the busiest emergency departments in the state of California, with more than 100,000 annual visits, including more than 3000 traumas [10]. ARMC serves the population of the County of San Bernardino, the largest county by area in the contiguous USA and a region with a high incidence of injection drug use.

A search of the ARMC electronic health system (EHR) was conducted for adult patients diagnosed with wound botulism between 2005 and 2020, using both the International Classification of Diseases 9th edition and 10th edition codes for wound botulism. Inclusion criteria were age > 18 years and had clinical symptoms suggestive of wound botulism (bulbar palsies and/or peripheral weakness). Confirmatory studies were performed on all patients, although five individuals either had insufficient blood sample acquisition or did not have the information in their charts. These five patients improved drastically after the administration of antitoxin and were thus included in the study as presumptive cases.

The EHR of each patient was reviewed to include data from ED encounters as well as subsequent inpatient care. The overall data included patients’ age, gender, symptoms at initial ED presentation, physical exam findings, history of illicit drug use, and results of ED diagnostic studies (i.e., labs, radiographs, EKGs). Additionally, patients’ clinical outcome data were collected, including hospital length of stay (LOS), mechanical ventilation, and percutaneous endoscopic gastrostomy (PEG) tube placement.

All statistical analyses were conducted using the SAS software for Windows version 9.4 (Cary, North Carolina, USA). Descriptive statistics were presented as frequencies and percentages for categorical variables, along with means and standard deviations or median and corresponding first and third quartiles for continuous variables. Wilcoxon rank-sum tests were conducted to assess whether the hospital length of stay was different between the categorical predictors. All statistical analyses were two-sided. *P* value < 0.05 was considered to be statistically significant.

## Results

A total of twenty-one patients were diagnosed with wound botulism at ARMC between 2005 and 2020. The average age was 38.48 (standard deviation = 8.41) years and seven of the patients were female. The most frequent symptoms during the initial ED encounter include history of heroin use followed by visible wound (*n* = 16, 76.2%), and symmetric motor weakness (*n* = 14, 66.7%). It is also notable that almost half of the patients had visible abscesses (*n* = 10, 47.6%) and more than half had ptosis (*n* = 12, 57.1%). The detailed symptoms list is presented in Table 1.

**Table 1:** Symptoms at initial ED presentation and diagnostic methods

	Frequency (***N*** = 21)	Percentage
**Symptoms at initial ED presentation**		
History of heroin usage	21	100.0%
Visible wound	16	76.2%
Symmetric motor weakness	14	66.7%
Ptosis	12	57.1%
Visible abscess	10	47.6%
Dysarthria	9	42.9%
Extraocular muscle palsy	8	38.1%
Surgical wound drainage	7	33.3%
Descending paralysis	6	28.6%
Infected abscess	6	28.6%
Signs of IV or subcutaneous heroin use	5	23.8%
**Diagnostic methods**		
Blood sample	11	52.40%
Unclear—antitoxin given	6	28.60%
CDC consultation—signs and symptoms	4	19.10%
Unclear	4	19.10%
EMG study	3	14.30%

*All other responses were either “no” or “not documented” by the physician

The physical examination results were analyzed to identify the most frequently noted clinical presentations associated with wound botulism in the ED. The results are presented in Table 2. The most frequent finding was generalized weakness (*n* = 20, 95%), followed by dysarthria/dysphonia (*n* = 14, 79%) and dysphagia (*n* = 15, 71%). All other findings occurred in less than 50% of patients.

**Table 2:** Physical examination findings at emergency department presentation

	Frequency (*N* = 21)	Percentage
Generalized weakness	20	95.20%
Difficulty swallowing	15	71.40%
Voice/speech problems	14	66.70%
Respiratory difficulty	12	57.10%
Double vision/diplopia	11	52.40%
Extremity/wound pain	9	42.90%
Shortness of breath	8	38.10%
Fever	7	33.30%
Blurry vision	6	28.60%
Sore throat	5	23.80%
Vomiting or diarrhea	5	23.80%
Dizziness	5	23.80%

Physical examination findings as documented in emergency department charts

*All other responses were either “no” or “not documented” by the physician

The effect of mechanical ventilation and PEG tube placement on hospital length of stay was also analyzed. Table 3 presents the results. Patients who were on mechanical ventilation had statistically significantly longer hospital LOS (median) compared with patients without mechanical ventilation (32 days vs 7 days, *p* < 0.001). Similarly, patients with PEG tube insertion had statistically significantly longer hospital LOS (median) compared with patients without PEG tube insertion (48 days vs 17 days, *p* < 0.001).

**Table 3:** Hospital LOS by mechanical ventilation and PEG tube status

	Hospital LOS	*P* value
**MV**		**< 0.001**
Yes	32 (22, 48)	
No	7 (3, 11.25)	
**PEG tube**		**<0.001**
Yes	48 (42, 61)	
No	17 (7, 26)	

*MV* mechanical ventilation, *PEG* percutaneous endoscopic gastrostomy

## Discussion

The natural progression of wound botulism varies from mild and insidious to severe and rapidly progressive. Seven strains of *C. botulinum* exist (A-G), but only A and B strains have been found to be associated with wound botulism secondary to injection drug use [11]. Type A is believed to be the most potent strain and causes the most prolonged disease course [4]. They are all gram positive, anaerobic rods with subterminal spores that thrive in the anaerobic conditions created by subcutaneous abscesses. *C. botulinum* produces neurotoxin that inhibits acetylcholine release by binding irreversibly to the presynaptic terminal [6, 12]. The resulting clinical syndrome is defined by symmetrical cranial nerve palsies [9, 13]. Mouth dryness, blurry vision, and double vision are reported as earliest neurologic complaints. More severe cases will progress to dysphagia, dysarthria, dysphonia, and peripheral muscle weakness. Furthermore, in its most severe form, a descending flaccid paralysis progresses to involve the respiratory muscles and causes neuromuscular respiratory failure [1, 4]. Notably, patients do not demonstrate any sensory deficits [14]. Recovery is often lengthy and requires generation of new neuromuscular connections [12].

The differential diagnosis should include wound botulism for any patient with a history of BTH use, presenting with generalized weakness and/or bulbar palsies. In addition, it is suggested that ED physicians rapidly request antitoxin, as well as investigating other possible etiologies such as the Miller-Fisher syndrome (MFS) variant of Guillain-Barre syndrome (GBS), myasthenia gravis, stroke syndromes (particularly basilar stroke), Lambert-Eaton Syndrome (LES), tick paralysis, or other generalized neuropathies [9, 15]. Emergency physicians may often have LES or the MFS variant of GBS as their top two differentials, so it is essential for the clinician to differentiate these conditions from botulism. In both botulism and LES, neurotransmitter release is inhibited at the presynaptic motor end plate. However, LES is due to antibodies against presynaptic voltage-gated calcium channels, and these highly specific antibodies can be detected in 85% of LES patients [16]. The MFS variant of GBS is in the differential due to its similar cranial nerve palsies, but it typically causes a noteworthy increase in CSF protein, which can help differentiate it from botulism [2]. Due to similar etiologies, it is imperative that a thorough evaluation be initiated to differentiate these diseases. However, in the case of wound botulism, these ancillary studies, such as serum electrolytes, hemoglobin, hematocrit, computed tomography (CT) of the brain, lumbar puncture, and other diagnostic modalities as indicated, will often be unremarkable [6].

Although neuromuscular transmission studies are often more sensitive, toxin assays are a standard part of diagnostic evaluation in patients suspected of having wound botulism [15]. Serologic testing for wound botulism requires a laboratory with adequate testing capabilities. Serum toxin assays have been reported to have sensitivities as low as 33-44%, whereas neuromuscular transmission studies have been shown in some reports to be diagnostic in up to 100% of cases [2, 15]. Nevertheless, these studies are not suitable for acute diagnosis due to delay in obtaining results. As a consequence, efforts to find a rapid diagnostic tool have shown some promise in creating a sensitive and specific test in PCR-based assays, immunoenzymatic assays, or bead-based suspension array [17].

Treatment of wound botulism with the equine-derived heptavalent (types A-G) or trivalent (types A, B, and E) antitoxin is aimed at interrupting the neurologic progression of the disease and mitigating the duration of ventilatory failure in those who are severely afflicted [6, 18]. The antitoxin neutralizes toxin that has not yet bound to nerve endings, which underscores the importance of early initiation of treatment. Treatment is complete after one dose because one vial generally contains more than enough antibodies to saturate unbound circulating toxin [9]. Additionally, the half-life of the antibodies is 5-8 days. Although antitoxin can be life-saving, clinicians must be aware that 21% of patients receiving antitoxin administration will demonstrate some degree of serum sickness reaction [15]. In severe cases, abscesses must also be drained properly to eradicate the source of bacterial toxins [19].

The morbidity and mortality of wound botulism can be drastically reduced if clinicians are adept at recognizing its clinical predictors. In addition, early recognition of wound botulism and antitoxin administration is critical to minimizing morbidity and mortality [7]. A high clinical suspicion along with a diagnostic and treatment protocol would help accelerate the process [7]. We noticed several diagnostic clues upon presentation which can assist in rapid identification and risk analysis for these individuals. Of note, everyone in this study presented with weakness or fatigue and a history of skin popping. Other common complaints included bulbar palsies (visual changes, difficulty swallowing, vocal changes), shortness of breath, and gait disturbances that were noted in prior reports [4]. Common physical exam findings included skin wounds, extremity weakness, ptosis, extraocular muscle abnormalities, and hypertension [4]. It is important to know that the process of obtaining botulinum antitoxin can be very challenging and time consuming. The local and/or state public health department must first be contacted, and then they contact the CDC. The CDC then arranges delivery of the antitoxin to the requesting hospital using their regional cache of the antitoxins. ED physicians are often unfamiliar with this process, which further prolongs time to administration of the antitoxin.

The generalizability of our findings may be limited. Like many retrospective reviews, the data collected in this study is limited by inconsistent documentation and recording bias. Efforts have been applied to thoroughly review EHR to ensure data accuracy.

## Conclusion

Wound botulism is a potentially fatal disease with rapid progression. It is therefore important to swiftly perform an evaluation, make the diagnosis, and initiate treatment. This process often begins in the emergency department, and as a result, emergency physicians must have a high clinical suspicion for wound botulism and be able to recognize early signs and symptoms of the disease.

## Data Availability

Availability of data and materials were available by contacting the corresponding author.
